# Impact of body image and perceived stigmatization on the psychological wellbeing of obese women in Kumasi metropolis, Ghana

**DOI:** 10.1371/journal.pone.0296061

**Published:** 2024-04-04

**Authors:** Harriet Afriyie-Adjimi, Anthony Kwabena Nkyi

**Affiliations:** 1 Counselling Centre, Kwame Nkrumah University of Science and Technology, Kumasi, Ghana; 2 Department of Guidance and Counselling, Faculty of Educational Foundations, College of Education Studies, University of Cape Coast, Cape Coast, Ghana; National Cheng Kung University College of Medicine, TAIWAN

## Abstract

Obesity is found to have a significant impact on body image perception and overall well-being. This study examines the impact of body image and perceived stigmatization on the psychological wellbeing of obese women in Kumasi metropolis, Ghana. A sample of 231 obese women was selected from health shops and some fitness centers using snowballing technique (purposive, snowballing technique and convenience). The study employed the descriptive survey design and made use of both descriptive and inferential data analysis approaches. The body shape questionnaire BSQ-34, the inventory of the Stigmatization Situation (SSI) and finally, the psychological well-being tools were used. Also, frequency distributions mean, and standard deviation, Pearson correlation coefficient and simple linear regression analysis were employed using SPSS version 23. Our findings indicated that obese women in the Kumasi metropolis were significantly satisfied with their body image. This is a true reflection of their higher self-esteem and standard of living. The body image and perceived stigmatization on the psychological wellbeing of the obese do have some counselling implications. Counselors, nutritionists, and clinical psychologists address specific schemes such as binge eating, dieting, and exercising to build the self-esteem of obese women.

## Introduction

Obesity is described as an unhealthy or excessive buildup of fat that is detrimental to one’s health. According to [1], over 1.9 billion people of 18 years of age and above were overweight. In sub-Saharan Africa (SSA) there has been an increase in obesity rates since 1990. West Africa, for instance, has obesity prevalence increasing from 2.6% to 7% between 1990 and 2015 [2]. Body image varies by gender, and while men usually prefer muscular bodies, women are more comfortable when they are slender. According to [3], males appear to underestimate their body size and are less concerned with weight regulation, while females have a tendency to exaggerate their body size. [4] found that obese women report a higher body image dissatisfaction. The Ghana Demographic and Health Surveys (GDHS) from 1993 to 2014 reported an increasing prevalence of obesity among Ghanaian women (15–49 years) from 3.4% to 15.3% (GSS, GHS, and ICF International, 2015).

Ghana has shown limited progress towards achieving the diet-related non-communicable disease (NCD) targets. 19.3% of adult women (aged 18 years and over) and 5.6% of adult men are living with obesity. Ghana’s obesity prevalence is lower than the regional average of 20.8% for women and 9.2% for men [5].

[6] indicates that there is evidence available that supports a high and rising prevalence of overweight and obesity among Ghanaian adults, and this is a significant public health issue. This has implications on current and future population health; the burden of chronic diseases and of health care spending can be enormous for a country that is still battling many infectious and parasitic diseases.

The issue of weight stigmatization also remains a challenge with the prevalence of obesity. In Ghana [7] reports that some obese women were stigmatized with name callings such as ‘*okeseɛ*’, ‘*obolo*’, *‘maame agbo*’. These are local nicknames which literally mean fat person. According to [7], participants found these nicknames very insulting. [8] indicated that obese Ghanaian women were more dissatisfied with their weight than normal weight women. Many women expressed unhappy sentiments about how being overweight has affected their lives in terms of their health, work life and social life. It has caused many to be anti-social and has affected their productivity at work, since they are always tired. The Ghana Demographic and Health Survey (GDHS) findings revealed a high prevalence of overweight/obesity (30.7 percent) among women in the Ashanti region [9]. The overweight/obesity rate was estimated to be 26% in Kumasi Metropolitan District Health Directorate (KMDHD) in 2008, specifically in Kumasi, the regional capital.

Body image dissatisfaction is predicted to be widespread as the incidence of obesity increases. The body image has also been defined as a subjective acceptance with one’s body and is multidimensional, concentrating on how much one weighs, the extent to which people approve their body size and their shape [10]. Perception of a person’s body size can be positive or negative and may be considered as accurate or inaccurate (an overestimation or underestimation of one’s body) or a feeling of being pleased or disappointed with one’s body [11]. People may call for these changes because they are not happy with how they look, and these could result from all kinds of distortions, such as how we feel, exposures, experiences and behaviors of parents and society as a whole [12].

[13], opined that the perception of body image has a great influence on psychological well-being. Obese people tend to feel stigmatized, and this feeling continues to affect their psychological well-being. Weight-based stigma is the societal rejection and devaluation of those who do not conform to the adequate body weight and shape of the prevailing social norms [14]. Weight-based stigma has some negative psychological consequences, such as low body confidence, self-esteem issues, anxiety, and depression [15] and these psychological issues can prevent obese people from living to their full potential [16]. There is the likelihood that obese people may be experiencing some psychological issues due to how they perceive themselves and the perceived stigmatization they may experience from their environs.

After a survey of older West African women, [17] indicated that almost half of the participants wanted a slimmer body size. According to [15], obesity could lead to stigmatization, and stigmatized overweight people who internalize these normative controllability values are more likely to become anti-social and experience psychological distress. Researchers, such as [17] as well as [18] believe that little is known about the psychological effect of body image on the Ghanaian woman. For instance, there is the likelihood that a number of Ghanaian women are unhappy in their marriages and relationships because their male counterparts are unhappy with their body sizes. This could probably be due to the perception that being slim is more attractive and better. Against this background the current study sought to explore the impact body image and perceived stigmatization on the psychological wellbeing of obese women in the Kumasi Metropolis, Ghana.

Our first objective was to determine the level of body image dissatisfaction among obese women. our second objective was to examine the level of perceived stigmatization among obese women. Our third objective was to examine the state of psychological well-being of obese women.

First, we hypothesize that there would be statistically significant relationship between body image and perceived stigmatization of obese women; second, we hypothesized that there would be statistically significant influence of body image on the psychological well-being of obese women; we further hypothesized that there would be statistically significant influence of weight-based stigma on psychological well-being of obese women.

## Methods

### Study area

Kumasi, a vibrant metropolis in the Ashanti region, spans from 6.350N to 6.400S latitude and 1.300W to 1.350E longitude, with an elevation of 250 to 300 meters above sea level. Covering approximately 214.3 square kilometers, it is one of thirty administrative districts in the region. Kumasi shares borders with Kwabre East to the north, Afigya Kwabre to the west, and Asokore Mampong, Atwima Kwanwoma, and Atwima Nwabiagya to the east. To the west lies Ejisu-Juaben, and to the south, it borders Bosomtwe (Ghana Statistical Service, 2014).

The metropolis comprises Sub-Metropolitan District Councils, including Asokwa, Suame, Bantama, Kwadaso, Manhyia, Oforikrom, Subin, Tafo, and Nhyiaso contributing to Kumasi’s diverse urban landscape (Ghana Statistical Service, 2014). Known for its robust infrastructure and health facilities, Kumasi is a hub of development and communal well-being in the Ashanti region.

### Demographics

Kumasi Metropolitan (or metro, for short), a vibrant city in Ghana, has a population of 443,981, according to the 2021 Census, with 213,662 males and 230,319 females. The age distribution shows a significant youth population, with 126,080 in the 0–14 years group and a large number of young adults in the 10–29 years range. The majority, 299,810 people, fall in the 15–64 years bracket, indicating a sizable working-age population. The city also has a notable senior population, with 18,091 individuals aged 65 and above. Overall, Kumasi Metro’s demographics depict a diverse and dynamic community with potential for growth and cultural richness.

Kumasi metropolis has nine (9) sub metros, hence the researcher selected one health shop and one fitness center (Gym) per each sub metro. In this study, 384 participants were chosen, focused on health shops and fitness centers in Kumasi, and utilizing multi-sampling techniques to ensure representation across Sub-Metropolitan District Councils. The sample size was evenly split between health shops and fitness centers (192 participants each) due to an unknown distribution of obese individuals.

Kumasi’s fitness centers, operating from 5 am to 9 pm, extend beyond exercise, serving as social hubs. These facilities have diverse exercise equipment, offer activities that promote physical fitness and foster a community atmosphere. There are others with peculiar services that provide extra amenities like massage and spa treatments. Kumasi residents display dedication during peak hours (5 am to 9 am and 5 pm to 9 pm). Natural health food stores used in this study open from 8 am to 5 pm, are spread across districts, and specialize in health foods and organic products. These stores cater to unique dietary needs, offering items like seeds, nuts, supplements, superfoods, and herbs. Some stores provide additional resources such as health coach and nutrition/dietetic services and assisting customers in making informed choices for overall well-being.

### Research design

The study was designed as a cross-sectional investigation, aiming to capture a snapshot of the relationships between body image, perceived stigmatization, and psychological well-being among obese women in the Kumasi Metropolis at a specific point in time. The study employed the descriptive survey design and made use of both descriptive and inferential data analysis approaches the focus was on assessing the prevalence of these variables within the target population. Data collection was conducted through surveys and interviews to gather information on participants’ body image perceptions, experiences of stigmatization, and psychological well-being. By adopting a cross-sectional approach, the study aimed to provide valuable insights into the current state of these factors and their potential associations.

### Population

The population is clearly identified as obese women in the Metropolis of Kumasi. The target population is obese women, and the accessible population consists of those obese women who visit specific areas chosen for the study. The areas selected for the study include health shops and gym or fitness centers in Kumasi.

The fitness centers were chosen because majority of its customers, who are obese, visited the place looking to lose weight through exercise. With respect to the health shops, a number of their customers also visited because of obesity-related conditions, typically, people with hypertension, diabetes, protruding bellies etc. They hoped to get supplements and other organic foods to cure their diseases or boost their health. On the whole, these categories of people were selected because the majority of people who visit these places were a perfect fit for the target population

### Inclusion criteria

1. **Obese Women:** The study includes obese women who have visited the designated premises in Kumasi within the past week, month, or year.
2. Females above 18 years old.

**Exclusion criteria** include Obese males and female minors were excluded from the study; People with physical deformities, because of the difficulties in getting accurate anthropometric measurements, and those with mental impairment with inability to understand and answer questions.

### Sample and sample size

The population of obese individuals in the Kumasi Metropolis who visited the selected premises for the study was undefined or unknown due to the inadequate records of client visits. Considering that the study population was unknown, the study relied on the Cochran formula for calculating sample size.

This formula allowed the optimal sample size to be determined based on the desired accuracy level, the confidence level, and the approximate proportion of the population with the target attribute. In this circumstance with large population, Cochran’s formula was considered suitable.

The formula for Cochran:

$$n_{0}=\frac{Z^{2}pq}{e^{2}}=\frac{{\left(1.96\right)}^{2}Χ(0.5)(0.5)}{{\left(0.05\right)}^{2}}=\frac{3.8416Χ0.25}{0.0025}=\frac{0.9604}{0.0025}=384\;\text{Obese}\;\text{Individuals}$$

1. N_0_ is desired sample size for a large population,
2. e is the desired level of precision (i.e. the margin of error),
3. p is the (estimated) proportion of the population which has the attribute in question,
4. q is 1 –p.

### Sampling procedure

From the 384 calculated sample size, 192 participants were sampled from the two main selected centers. The sample size was disproportionately shared among the two groups. This meant 192 participants from the health shops and 192 from the fitness centers. This equal distribution was necessary due to the difficulty in justifying any form of proportionate distribution as the population of obese individuals from the health shops and fitness centers (Gyms) were unknown. The study employed multi sampling. Multi sampling techniques were used because the researcher needed to employ various sampling techniques. First, obese individuals were selected from the two basic centers: the health shops and the fitness centers (Gyms) in Kumasi. Purposive sampling was then employed. [19] indicates that purposive sampling was appropriate when the researchers relied on their own judgments in selecting the units (people, case/organisations, events, pieces of data). One advantage of purposive sampling is that they can provide researchers with the justification to generalize from the sample that is being studied. Thus, obese women were purposively chosen because these centers were places where they were more likely to be found. These health centers in Kumasi are often visited by obese individuals seeking professional advice and solutions to their weight problems. Third, using the snowballing technique, the fitness centers and the health shops were located beginning with identifiable points. The researchers identified obese individuals who visited these places for assistance and they, in turn, directed the researchers to other places where they were likely to find individuals with characteristics similar to what the researchers were interested in. Finally, the individual participants were further selected from the identified centers through the convenience sampling method. With the convenience sampling, the researchers contacted the participants as and when they visited the gym and the health shops. [20] defined convenience sampling as a method that relies on data collection from population members who are conveniently available to participate in a study. It is a sampling method that involves getting participants wherever you can find them and, typically, wherever it is convenient. All subjects are invited to participate. Convenience sampling was appropriate because it could be applied to gain initial primary data regarding specific issues, such as perception of body image, stigmas and their psychological well-being.

### Measures

**Demographic variables** include age, education, marital status, and religion.

There were three parts of the questionnaire: anthropometric results, the body shape questionnaire, and the inventory of the stigmatization situation. The instruments used for the analysis were all adopted.

To assess the individual’s Body Mass Index (B.M.I.) status, anthropometric data were taken. This determined the obesity status of the person. With a stadiometer, the individual’s height was taken to the nearest 0.1 cm. The weight was also taken to the nearest 0.1 kg on a weighing scale. The body mass index was measured and rated according to W.H.O. standards by dividing the weight in kg by the square of the height in meters. In an underweight person, B.M.I is <18.5; normal weight BMI is 18.5–24.9; an overweight person’s B.M.I. is 25–29.9, and an obese person’s B.M.I. is >30. (1998-W.H.O.).

**The Body Shape Questionnaire (BSQ)** instrument is a self-report questionnaire developed to measure concern about body shape and appearance in the normal population and clinical population. The Body Shape Questionnaire (BSQ) was developed by [21] to measure concern about body weight and shape experienced by persons’ related body image problems. This instrument consists of 34 questions that ask participants how they feel about their performance over the past four weeks. The BSQ instrument has six Likert scale points, namely 1 (never), 2 (rarely), 3 (sometimes / sometimes), 4 (often), 5 (very often), and 6 (always). This instrument can be completed in 10 minutes. Based on the total score, the assessment of the instrument is divided into four levels, namely no attention (total score ≤ 80), mild attention (total score 81–110), moderate attention (total score 111–140), and severe attention (score total> 140). Cronbach’s Alpha for reliability for BSQ is 0.928.

**Stigmatizing Situations Inventory (SSI)** is a 50-item measure assessing participants’ frequency of experiencing stigmatizing situations [22]. The SSI has been commonly used in community populations to measure the frequency of experiencing 50 stigmatizing situations. It assesses weight stigmatization over time. This questionnaire assesses the stigmatizing experiences associated with being overweight that may have occurred to a subject at least once in his/her lifetime. Each item describes a stigma encounter and asks the respondent to rate the intensity of the experience on a 10-point Likert-type ranging from 0 (never) to 9 (frequent/daily). The SSI includes eleven subscales that highlight various types of weight-related stigmatizing experiences. These subscales include comments from children; others making negative assumptions about you; physical barriers; being stared at; inappropriate comments from doctors; nasty comments from family; nasty comments from others; being avoided, excluded, ignored; loved ones embarrassed by your size; job 15 discrimination; and being physically attacked. In several studies using the SSI, a higher level of stigma attitudes has been linked to higher levels of depression and anxiety, lower self-esteem and quality of life, greater body dissatisfaction, and higher binge eating. The result is obtained by calculating the average of all responses. The higher the score, the greater the number of exposures to stigmatizing experiences. This inventory showed excellent internal consistency and good validity (Cronbach’s alpha = 0.95).

The 18-item version of **Ryff’s Psychological Well-Being Scale** [23] is a self-report instrument that comprises 18 items measuring six dimensions of psychological wellbeing: autonomy, environmental mastery, self-acceptance, personal growth, positive relations with others, and purpose in life. It is a 7-point Likert with responses ranging from 1 = strongly agree; 2 = somewhat agree; 3 = a little agree; 4 = neither agree nor disagree; 5 = a little disagree; 6 = somewhat disagree; 7 = strongly disagree. The Autonomy subscale items are Q15, Q17, Q18. The Environmental Mastery subscale items are Q4, Q8, Q9. The Personal Growth subscale items are Q11, Q12, Q14. The Positive Relations with Others subscale items are Q6, Q13, Q16. The Purpose in Life subscale items are Q3, Q7, Q10. The Self-Acceptance subscale items are Q1, Q2, and Q5. Q1, Q2, Q3, Q8, Q9, Q11, Q12, Q13, Q17, and Q18 should be reverse scored. Reverse-scored items are worded in the opposite direction of what the scale is measuring. The formula for reverse-scoring an item is: ((Number of scale points) + 1)–(Respondent’s answer) For example, Q1 is a 7-point scale. If a respondent answered 3 on Q1, you would re-code their answer as: (7 + 1)– 3 = 5. In other words, you would enter a 5 for this respondents’ answer to Q1. Therefore, the total score is in the range of 18–108, with higher scores representing greater well-being. To calculate subscale scores for each participant, sum respondents’ answers to each subscale’s items. Higher scores mean higher levels of psychological well-being. The internal accuracy varies from 0.86–093 [24].

### Confirmatory factor analysis

This section contains the results on the validation of the body shape questionnaire, psychological well-being and stigmatizing situation inventory (Table 1). Data were collected from 100 obese women in the Kumasi Metropolis. Confirmatory factor analysis (CFA) was performed using Smart-PLS.

**Table 1:** 

Constructs	Items	Loadings	rho_A	CR	AVE
**Stigmatizing Situation Inventory**					
1. Comments from children			.772	.840	.518
	C1	.601			
	C6	.786			
	C7	.805			
	C19	.799			
	C32	.572			
2. Comments from doctors			.802	.871	.629
	C2	.857			
	C16	.763			
	C22	.686			
	C23	.855			
3. Comments from family			.774	.839	.511
	C3	.624			
	C4	.772			
	C5	.757			
	C24	.692			
	C44	.720			
4. Comments from others			.759	.835	.563
	C10	.835			
	C30	.687			
	C33	.623			
	C43	.832			
5. Negative assumptions			.752	.854	.663
	C28	.788			
	C29	.883			
	C42	.767			
6. Physical barriers			.834	.859	.443
	C8	.680			
	C15	.702			
	C35	.706			
	C36	.377			
	C37	.607			
	C38	.767			
	C39	.827			
	C40	.555			
7. Avoided, excluded, ignored			.789	.668	.603
	C13	.710			
	C18	.898			
	C45	.785			
8. Being stared at			.971	.969	.397
	C14	.809			
	C47	.763			
	C49	.820			
	C50	.719			
9. Loved ones			.781	.858	.670
	C25	.868			
	C26	.852			
	C27	.729			
10. Job discrimination			.684	.791	.575
	C11	.881			
	C34	.448			
	C41	.865			
11. Physically attacked			.868	.875	.480
	C9	.407			
	C12	.786			
	C17	.634			
	C20	.849			
	C21	.881			
	C31	.710			
	C46	.629			
	C48	.503			
**Psychological Well-being**					
12. Autonomy			.789	.668	.603
	D15	.305			
	D17	.936			
	D18	.917			
13. Environmental mastery			.714	.629	.512
	D4	.160*			
	D8	.839			
	D9	.897			
14. Personal growth			.730	.593	.580
	D11	.909			
	D12	.861			
	D14	.414			
15. Positive relations			.189	.036	.377
	D6	.451			
	D13	.767			
	D16	.583			
16. Purpose in life			.570	.003	.428
	D3	.898			
	D7	.466			
	D10	.508			
17. Self-Acceptance			.654	.533	.566
	D1	.833			
	D2	.874			
	D5	.489			
**Body Shape Questionnaire**			.960	.956	.401
	E1	.647			
	E2	.778			
	E3	.577			
	E4	.622			
	E5	.644			
	E6	.528			
	E7	.579			
	E8	.550			
	E9	.207*			
	E10	.722			
	E11	.708			
	E12	.710			
	E13	.626			
	E14	.716			
	E15	.734			
	E16	.607			
	E17	.529			
	E18	.528			
	E19	.685			
	E20	.825			
	E21	.757			
	E22	.377			
	E23	.491			
	E24	.768			
	E25	.595			
	E26	.578			
	E27	.607			
	E28	.492			
	E29	.680			
	E30	.334			
	E31	.773			
	E32	.656			
	E33	.808			
	E34	.607			

*Items deleted

**Fig 1:**
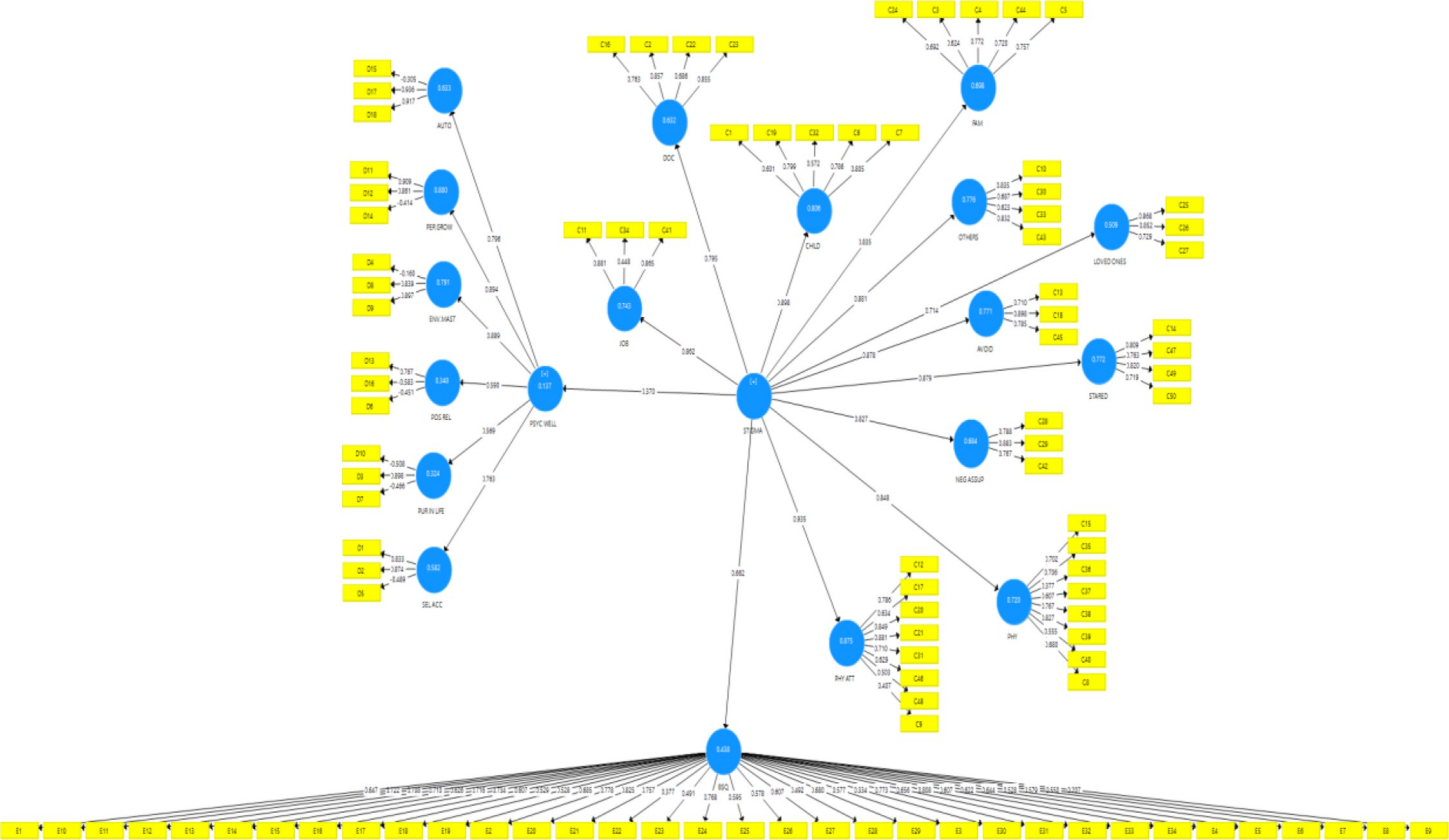
Measurement model for stigmatizing-situational inventory, body shape questionnaire and psychological wellbeing.

As indicated in the table above, two items (D4 and E9) had factor loadings below .30 and, therefore, were discarded. In all, 90 items were deemed valid for the data collection. The AVEs for all the dimensions were resonable; hence, convergent validity was achieved (Tables 2 and 3 and Fig 1).

**Table 2:** 

Construct	1	2	3	4	5
Autonomy					
Environment	.279				
Personal	.330	.219			
Positive relations	.862	.816	.649		
Purpose in life	.500	.772	.728	.885	
Self-acceptance	.788	.877	.548	.660	.718

**Table 3:** 

	1	2	3	4	5	6	7	8	9	10
Child										
Doc	.431									
Fam	.646	.733								
Other	.333	.892	.646							
Neg	.894	.875	.857	.828						
Bar	.550	.768	.633	.716	.758					
Avoid	.352	.816	.847	.675	.459	.887				
Stared	.790	.881	.833	.538	.545	.843	.697			
Loved	.695	.502	.805	.767	.840	.594	.874	.662		
Job	.545	.765	.795	.653	.890	.716	.246	.772	.328	
Phys	.623	.845	.804	.743	.853	.845	.675	.828	.707	.743

According to [25], to achieve discriminant validity, the HTMT values must be below .90. For the discriminant validity, all the HTMT values for the dimensions of stigmatising situational inventory, body shape questionnaire and psychological wellbeing were below .90; hence discriminant validiy was achieved. Further, the internal reliability was estimated using the rho_A, and most of the coeffiecients were above .70. In all, 90 items were used for the data collection. presents the measurement model of the stigmatising situational inventory, body shape questionnaire and psychological well-being.

### Validity and reliability of data collection instrument

Validity establishes the extent to which the data collection instrument can collect the data it is supposed to collect. In the development of a research design, the researcher needs to be sure that empirical findings that reflect the reality of situations are collected [26]. Pretesting is fundamentally important in determining the strengths and weaknesses of the instrument before the actual data collection. It also allows for rectification on invasive or intrusive questions [27]. Consistent with this view, the researcher pretested the instrument with 50 respondents in the study area, to examine the understanding of the respondents and the possibility of gathering the required information with the instrument. The outcome of this activity enabled the researcher to review and restructure the instrument to reflect this goal. The questionnaire was also reviewed by expert colleagues, and their inputs were gathered and factored in the restructuring to obtain both content and face validity of the instrument.

### Ethical clearance

The Institutional Review Board of the University of Cape Coast approved the study (ID-UCCIRB/CES/2020/81) before the data collection. Permission was sought from the respective managements of the various selected premises for the study to be carried out. The right to privacy, voluntary participation, no harm to participants, anonymity, and confidentiality were the ethical concerns adhered to. Participants signed an informed consent form before responding to the questionnaire. Their rights for involvement were respected. Clients and staff of the selected premises were also informed about how this exercise could disrupt normal activities at the various premises.

### Data collection procedure

The researchers were assisted by three research assistants who received training before the data collection. One health worker was employed to undertake the anthropometric data. Permission was sought from the management of the various centers to enable us carry out the study. Participation was voluntary through informed consent from the participants. The researchers clarified the purpose of the study to the participants and assured them of confidentiality and anonymity. Questionnaires were administered to the participants at the various selected locations from the November 13, 2020, to December 16, 2020, during the institutions’ working hours. Participants took 30–40 minutes to complete the questionnaire. All safety and health protocols were observed during the data collection process due to the coronavirus (COVID-19). A total of 231 participants completed the survey.

### Data processing and analysis

Data were imported and analysed using SPSS version 23 for Windows (IBM Corp., Armonk, NY). at a significance level of 5%. (0.05). After data cleaning, data were summarized using both descriptive and inferential statistics. Continuous variables (Age, Height, Weight, Waist circumference, Hip circumference, and Body mass index) were summarized with means and standard deviation, while categorical variables (educational level and marital status) were presented as frequencies and percentages. All the three research questions were analyzed using the mean and standard deviation. One sample t-test was used. The association between Body Image Satisfactions and perceived weight-related stigma was analyzed using Pearson Correlation Coefficient. Simple linear regression was used to analyse hypothesis two to determine the significant influence of body image on the psychological wellbeing of the obese. Hypothesis three was analyzed using simple linear regression. The measurement items were validated through exploratory factor analysis and reliability checked through Cronbach Alpha analysis.

## Results

The demographic characteristics of the participants are presented as the age, educational level, anthropometric measures, body mass index, and marital status of the respondents are among the demographics addressed (Table 4).

**Table 4:** 

Demographic participants		Frequency			Percentage	
**Educational level**						
None		5			2.2	
JHS/**JSS**		4			1.7	
SHS/VOC./TECH.		93			40.3	
Tertiary		129			55.8	
**Total**		231			100	
**Marital Status**						
Single		120			51.9	
Married		111			48.1	
**Total**		231			100	
**Descriptive statistics**	**Mean**		**SD**	**Minimum**		**Maximum**
Age	40.35		9.35	23.00		67.00
Height	158.47		6.96	146.00		180.00
Weight	107.89		12.20	83.00		135.00
Waist circumference	43.15		3.66	38.00		54.00
Hip circumference	50.34		4.40	40.00		65.00
Body mass index	42.94		3.73	35.92		56.89

The results reveal that the majority of the participants (129) have tertiary education representing 55.8% of the total participants. (Table 4) Majority of participants (120) were unmarried women that represent 51.9% of the participants. The average age of participants is 40.35 years. This implies that the majority of participants were between middle and late adulthood.

### Anthropometric measurement

The results show that the average height of the participants among obese women is 158.47 cm with a standard deviation of 6.96 cm. The standard deviation shows that there were less variations in the heights of the sampled obese women. The average weight of the participants among obese women is 107.90 kg with a standard deviation of 12.20 kg. Standard deviation is low suggesting that the average weight is representative. Again, the standard shows less variation in the weight of the sampled obese women. The average hip circumference of the respondents among obese women is 43.15 cm with a standard deviation of 3.66 cm. Standard deviation is low suggesting that the average waist is representative. Again, the standard shows that there were less variations in the waists of the sampled obese women. The average waist circumference of the respondents among the obese women is 50.34 cm with a standard deviation of 4.40 cm. Standard deviation is low suggesting that the average waist circumference is representative. Again, the standard shows less variation in the average waist circumference of the sampled obese women.

### Body mass index

Finally, the average body mass index (BMI) of the sampled women is 42.94 kg/m with a standard deviation of 3.73kg/m. Since the minimum BMI and average BMI are all above 30kg/m, it implies that women sampled for the study are obese [1]. Standard deviation is low suggesting that the average BMI is representative. Again, the standard shows that there is less variation in the BMI of the sampled obese women.

Research question one was to determine the level of body image dissatisfaction among obese women. Results indicate that generally obese women in the Kumasi Metropolis were significantly satisfied with their body image. (*M* = 86.95, *SD* = 26.06). The result reveals that the level of Body Image Dissatisfaction is statistically low (Table 5). The Table 5 presents the results of the mean and standard deviation of Overall Level of Body Image Dissatisfaction among obese women in the Kumasi Metropolis.

**Table 5:** 

Variable	Mean	SD
**Overall level of Body Image Dissatisfaction**	86.95	26.06

M = mean; SD = standard deviation.

In research question two, the objective was to investigate the level of perceived stigmatization among obese women. The results of the mean and standard deviation of the general level of perceived weight-related stigma among obese women in the Kumasi Metropolis (Tables 6 and 7).

**Table 6:** 

Variable	Mean	SD
**General level of Body Image Dissatisfaction**	36.29	45.619

M = mean; SD = standard deviation.

**Table 7:** 

Variable	Mean	SD
**Composite of Psychological Well-being**	56.16	11.79

M = mean; SD = standard deviation.

The results from Table 6 indicate that the perception of weight-related stigma is significantly low (M = 36.29, SD = 45.619). The result indicates that obese women in the Kumasi Metropolis perceived that they are not significantly stigmatized due to their body weight and size.

Research question three sought to find the level of psychological well-being among obese women in the Kumasi Metropolis. The results from Table 7 reveal a significant positive psychological well-being among obese women (M = 56.16, SD = 11.79).

**Fig 2:**
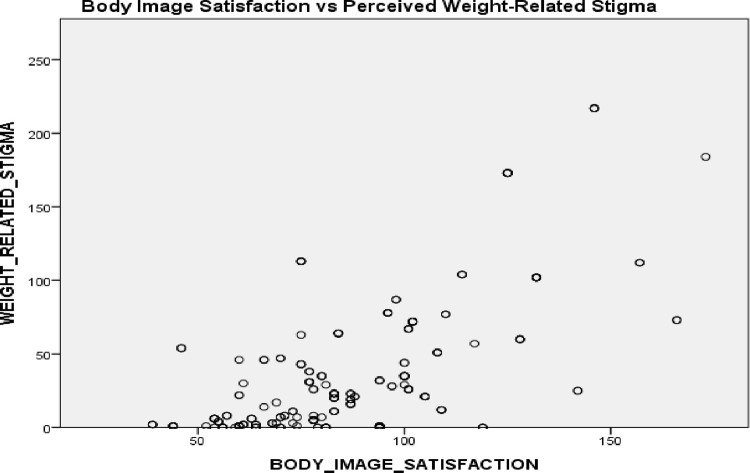
Scatter plot of linearity.

The objective of hypothesis one was to determine the relationship between body image and perceived stigmatization of obese women in Kumasi Metropolis. A statistical tool is useful in determining the relationship among variables with continuous data. The analysis aided in identifying the direction and degree of the relation among the variables under consideration. Before computing the correlation analysis, an assumption was tested to verify the Pearson Correlation Coefficient. Linearity among the variables was estimated using a scatter plot. Fig 2 presents the scatter plot normality test of the study variables.

The result of the linearity (linear relationship) as presented by the scatterplot indicated that there was a positive linear relationship between body satisfaction and perceived stigma (Fig 2). This was because the direction of the plots was tilted from left to right. The use of Pearson Correlation is supported by this finding and the continuous nature of the data. Table 8 presents the analysis of Pearson correlation coefficients of body satisfaction and perceived stigma.

**Table 8:** 

VARIABLES		Body image Satisfaction	Perceived Stigmatization
**Body Image Satisfaction**	Pearson Correlation	1	.659**
	Sig. (2-tailed)		.000
	N		231
**Perceived Stigmatization**	Pearson Correlation		1
	Sig. (2-tailed)		
	N		

** Correlation is significant at 0.01 level (2-tailed); N = sample size

The results showed a significant positive relationship between body image perception and perceived stigmatization (r = .659) at a 0.01 level of significance. The positive correlation between body image and perceived stigma implied that body image satisfaction was related to a decrease or absence of weight-based stigma. The magnitude of .659 shows that there is a strong correlation between the level of body image satisfaction and perceived stigmatization (Table 8).

The objective of hypothesis two was to determine the influence of body image on the psychological well-being of obese women. In this analysis, the independent variable is body image, and the dependent variable is psychological well-being (Tables 9 and 10).

**Table 9:** 

Model	Standardized Coefficients	Unstandardized Coefficients		t-value	p-value
	**Beta**	**B**	**Std. Error**		
(Constant)		57.534	2.714	21.203	.000
Body Image	- .035	-.016	.030	-.527	.599

Dependent Variable = Psychological Well-being t = t-statistic; p = Significant, p < 0.05

**Table 10:** 

Variables	*df*	Sum of Squares	Mean square	*F*	Sig.	*R*	*R* ^*2*^
Regression	1	38.777	38.777	.278	.599	.035	.001
Residual	229	31978.972	139.646				

M = mean; df = degrees of freedom; F = F-statistic

The results from Table 10 revealed that body image perception has no significant effects on the psychological well-being of obese women (β = -.035, p = .599). The above results indicate that the body image predicted less than 1% of variances in the psychological well-being of obese women (R^2^ = .001, t = .035, p = .599); however, this value was insignificant because the significant value (p-value) was greater than 0.05. The result implies that body image is insignificant at explaining variation in the psychological well-being of obese women.

Hypothesis three was to investigate the influence of weight-related stigma on the psychological well-being of obese women in the Kumasi Metropolis. A simple linear regression analysis was considered appropriate to analyze this hypothesis. In this hypothesis, the independent variable is weight-related stigma, and the dependent variable is psychological well-being. The results of the linear regression analysis are presented below (Table 11).

**Table 11:** 

	Standardized Coefficients	Unstandardized Coefficients					
Model	Beta	B	Std. Error	t	Sig.	R	*R* ^*2*^
(Constant)		55.531	.993	55.939	.000		
Weight-Related Stigma	.068	0.17	.017	1.025	.307	.068	.005

Dependent Variable = Psychological Well-being Significant, p<0.05

Table 11 indicates that, generally, perceived weight-related stigma does not significantly influence psychological well-being of obese women in the Kumasi Metropolis (β = .068, p = .307). Perceived weight-related stigma explained less than 1% variance of psychological well-being of obese women. This finding suggests that perceived weight stigma has no significant impact on the psychological well-being of obese women.

## Discussion

Obesity is found to have a significant impact on body image perception and overall well-being. The research question sought to identify the level of body image satisfaction among obese women. The results of the study revealed that obese women in the Kumasi Metropolis were significantly satisfied with their body image. The finding implied that irrespective of their body size and weight, obese women in the Kumasi Metropolis were generally satisfied with their body image. In terms of percentages, it was revealed that a majority of the women were slightly dissatisfied with their body image. The findings could be attributed to the preference of Africans for an average and fat body. Among Ghanaians, weight gain is equated to prosperity and good living. [2], demonstrated that there is a presumed cultural valuation of fatness as a sign of good health, fertility, wealth, and beauty. Similarly, [28] opined that whilst extreme obesity is undesirable among Ghanaian women, some level of weight gain is admirable and seen as good signs of living. In their study, [18] found that the prevalence of overweight/obesity among the women was 68.4%. The respondents preferred a large (overweight) body size. They associated large (overweight) body size with eating well, affluence and high social value. Though the overweight/obese respondents associated normal body size with health they preferred a large (overweight) body size. They concluded that Sociocultural ideals for body size override health reasons for the women’s preferred body size. This study shows that tackling the overweight/obesity problem solely from nutrition and health perspective may not be adequate.

This assertion justifies why obese women in the present study were generally satisfied with their body image. The findings of this study are inconsistent with that of [29] who explored body image perception among university students of Sharjah in the United Arab Emirates (UAE). Findings revealed that 81% (58.2% females) were significantly dissatisfied with their body image. The majority of the studies dealing with the current study topic found that body image dissatisfaction is higher among many groups of individuals. Whiles this is not certainly true for the findings of the current study, it could be asserted that the differences are due to the cultural beliefs, social environment, and the type of the participants who were recruited for the study.

The research question two was to identify the level of perceived weight-based stigma among obese women. Findings indicated that obese women in the Kumasi Metropolis perceived that they were not stigmatized due to their body image and size. The findings defy the assertion that weight-related stigma is prevalent in recent times [30]. It has been noted that weight-related stigma persists because people do not consider the genetic, illness, and injury factors related to obesity, but, instead, dwell strictly on extensive food consumption and sedentary lifestyle. The role of the media in projecting thin and slim body as desired body image also increases weight-related stigma in our society. In Ghana, obese people are popularly referred to as ‘*okeseɛ’*, ‘*obolo’*, *‘maame agbo’*. This name calling can hurt the self-esteem of obese people in general. However, in this study, weight-related stigma was not an issue among obese women in the Kumasi Metropolis. Inconsistent with our findings regarding weight-related stigma, [28] studied the views and experiences related to Ghanaian women being overweight. They revealed that overweight women were highly stigmatized.

The findings indicated that obese women had significant positive psychological well-being. The results may be that in the African context, having more weight and being a bit “fat” is seen as a sign of good living and better life. However, a study, by [31] which considered weight stigma and mental health among women, reported that the effect of weight-based discrimination on psychological well-being is highly contingent on social status. Specifically, the psychological consequences of discrimination on women are significantly greater relative to controlling for obesity status and self-rated health. These results suggest that, due to socio-economic status, weight stigma has an adverse impact on psychological well-being.

The results reported that body image had a significant positive relationship with weight-related stigma. This means positive relationship connotes that body image satisfaction is related to a decrease or absence of weight-based stigma. In other words, the less obese women are discriminated against, the higher their body image satisfaction. Similar to this finding, [32] observed a moderate positive relationship between body image and weight-related discrimination. [33] assessed the impact of weight labels on body satisfaction, weight-related stigma, and other variables among college women. Findings showed that weight labels and related stigma had a significant negative impact on body image satisfaction. This result connotes the existence of a relationship between body image and weight-related stigma.

The findings are that body image has no significant effect on the psychological well-being of obese women. They imply that the perception of being obese has no impact on the psychological health among women in the Kumasi Metropolis. These surprising findings defy the assertions of many studies that obesity is associated with psychiatric disorders. According to [34], the psychological impact becomes significant when obese women become increasingly worried about their body image and become preoccupied with weight loss. In this current study, obese women showed satisfaction with their body image. Based on these findings the results that body image perception did not influence participants make statistical sense. Consistent with these findings, [35] explored the relationship between psychological well-being and obesity among women with infertility problem.

Culture also plays a role in body image since it clearly shows differing criteria concerning attractiveness. [36] observed a different satisfaction level with ethnicity. Several studies have indicated the preference for larger body sizes by Africans, African Americans, and Caribbeans [37]. In Ghana, for instance, some cultures equate weight gain to a sign of prosperity and good living. [2, 38] showed that culturally, there was variation in how fatness is perceived, as a symbol of good health, fertility, wealth, and good appearance. An increase in post-marriage body weight status is perceived as a symbol of a good life and happiness, and willingness of the husband to take good care of the wife. [28] also confirmed that though extreme obesity was unwelcome by most Ghanaian women, some amount of weight gain was desired and seen as a sign of wealth and good care by a spouse.

Results of the study indicated that generally, perceived weight-related stigma does not significantly influence the psychological well-being of obese women in the Kumasi Metropolis. Based on the findings of this study obese women had positive psychological well-being and perceived that they are not stigmatized due to their body image or size. Consistent with this study, [31] indicate that weight-related stigma has no direct effect on the psychological well-being among women; rather, the influence of other factors, such as socioeconomic elements, makes the effect of body stigma and weight significant.

### Implications for counselling

Impact of body image and perceived stigma on the psychological wellbeing of the obese does have some counselling implications. The need for tailored intervention, such as individual, group, family counselling and Psychoeducation, have been shown in studies to be effective for a wide range of client concerns [39]. Both prevention and intervention strategies are needed to help women realize body image problems and to help professionals appropriately recognize the symptoms that may be overlooked. Counselling could result in behaviour change, successful experiences, and mastery for parties involved.

According to the study, majority of obese women in Kumasi were satisfied with their bodies. This is reflective in their high self-esteem and standard of living. As a result, they show little concern about how their body size impacts negatively on their significant others. As such, counsellors are to counsel and address specific behaviours (e.g., binge eating, dieting, and exercising) and to draw their attention to the obese so they don’t overlook the effects of their obesity on the important people around them.

The study, also, revealed some level of stigmatization obese women go through. Though majority do not experience this, there is the possibility that those who experience stigma have a range of unhealthy behaviours and cognitive distortions that could lead to symptoms of depression and low self-esteem [40–42]. Given this, Counsellors can help clients acknowledge and normalize the concept of body image dissatisfaction they experience. They can also create a supportive, collaborative counselling relationship through family and group counselling.

## Conclusion

The study was a quantitative survey and it investigated the influence of body image and perceived stigmatization on the psychological well-being of obese women in the Kumasi Metropolis

From the findings of the study, several conclusions are drawn. Firstly, obese women in the Kumasi Metropolis are not disturbed by their “plus size” body image, since many of the subjects (obese women) were significantly satisfied with their body image. Also, being an obese woman in the Kumasi Metropolis has no adverse effect on one’s psychological well-being, despite their body size. Lastly, body image satisfaction is related to a decrease or absence of weight-based stigma in obese women in the Kumasi Metropolis.

### Limitation

This study was limited in so many ways. Despite the fact that the sample size was determined using an appropriate and approved formula, the surge of the Covid’19 prevented the researcher from accessing certain premises, affecting the sample size. Additionally, due to the sensitive nature of the topic, the participants had to be persuaded before getting them to willingly answer the questionnaire.
